# PVP-40 mediated enhancement of mesophyll protoplast yield and viability for transient gene expression in black huckleberry

**DOI:** 10.1186/s13007-025-01471-9

**Published:** 2025-11-18

**Authors:** Sweety Majumder, Abir U. Igamberdiev, Samir C. Debnath

**Affiliations:** 1 St. John’s Research and Development Centre, Agriculture and Agri-Food Canada, St. John’s, NL Canada; 2 Department of Biology, Memorial University of Newfoundland, St. John’s, NL Canada

**Keywords:** *Vaccinium membranaceum*, Protoplast isolation, Enzymatic digestion, Transformation efficiency, Green fluorescent protein, Subcellular localization

## Abstract

**Background:**

Black huckleberry (*Vaccinium membranaceum*) is a native fruit species of high nutritional, medicinal, ecological, and economic value. The black huckleberries, abundant in bioactive compounds, offer significant antioxidants and anti-inflammatory effects and play a key role in maintaining wildlife and forest ecosystems. Despite its importance, protoplast isolation and gene editing have not been reported in this species. These techniques are essential for functional genomics and crop improvement, but the recalcitrant nature of this species, complex genome, and variable ploidy present significant challenges for cellular and molecular manipulation. This study aimed to establish a reliable protocol for efficient mesophyll protoplast isolation and transient gene expression in *V. membranaceum* using in vitro-grown leaves.

**Results:**

A systematic optimization of enzyme composition, osmotic concentration, antioxidant supplementation, and pH was undertaken to enhance protoplast yield and viability in *V. membranaceum*. The optimized enzymatic combination of 2% cellulase R-10, 1% hemicellulase, 1% Macerozyme R-10, and 1.5% pectinase facilitated efficient cell wall degradation while maintaining structural integrity. The inclusion of 0.6 M mannitol ensured osmotic stability, and 1% PVP-40 effectively suppressed phenolic oxidation, significantly improving protoplast viability. A near-neutral pH of 5.8 supported optimal enzyme activity without inducing cellular damage. Under these optimized conditions, 14 h enzymatic digestion produced 7.20 × 10⁶ protoplasts g⁻^1^ FW with 95.1% viability. Subsequent optimization of PEG-mediated transformation identified 40% PEG-4000 with 30 µg plasmid DNA as the most effective combination, achieving 75.1% transient expression efficiency. Nuclear localization of GFP-tagged proteins, confirmed by DAPI staining, validated the robustness of the optimized system.

**Conclusions:**

This study presents a standardized, PVP-40–enhanced protocol for mesophyll protoplast isolation with notable yield and viability in *V. membranaceum*, supporting efficient transient gene expression. The method provides a robust platform for functional genomics, gene editing, and biotechnological applications in this underutilized species and other related plant species.

## Introduction

*Vaccinium membranaceum* is a woody, perennial, deciduous shrub in the family *Ericaceae*, native to western North America, especially in Northwest British Columbia [1]. This tetraploid species, commonly known as mountain black huckleberry, produces fleshy, edible fruits rich in phenolic compounds and antioxidants that contribute to human health benefits [2, 3]. These fruits are moderately rich in antioxidants, anthocyanins, flavonoids, and phenolic compounds that contribute to their health benefits, including anti-inflammatory, cardioprotective, and anti-aging effects [3]**.** Although *V. membranaceum* is economically and ecologically significant, its traditional propagation methods are limited by low seed germination, weak root development, and high phenolic content. Additionally, its perennial nature and genetic variability impede its ability to meet the growing demands for climate resilience, consumer preferences, and socio-economic adaptability [4].

Additionally, genetic engineering has facilitated notable advancements in fruit crops by improving traits including tolerance to biotic and abiotic stresses, as well as fruit quality characteristics such as shape and ripening [5]. However, the genetic transformation and functional genomics of *Vaccinium* species remain particularly difficult due to their resistance in tissue culture, low transformation success rates, and the challenges posed by their recalcitrant nature [6]. This context highlights the need for alternative methods of genetic modification in *V. membranaceum*.

Protoplast isolation is a crucial technique in plant biotechnology, particularly for gene editing applications [7]. In woody plants, the complex secondary metabolism, rigid tissue architecture, and limited regenerative capacity of woody plants present significant challenges in the development of effective protoplast isolation and regeneration systems, which hinder the application of biotechnological breeding methods [8].

Effective transformation protocols are essential for genetic modification strategies [9]. To date, various techniques such as particle bombardment and *Agrobacterium tumefaciens*–mediated transformation of leaf explants and callus tissues have been successfully employed in the genetic modification of multiple fruit crops [6]. However, these methods are limited due to high equipment costs, reduced transformation efficiency, slow in vitro regeneration, and poor gene expression, hindering high-throughput functional gene analysis [10]. Protoplast-based transient transformation offers significant advantages over traditional methods, especially for functional gene analysis [11, 12]. Recently, transient protoplast transformation has been utilized in diverse plant species to analyze protein functions, including localization, interactions, stability, and degradation, and to support genetic advancements [13].

Protoplasts are plant cells lacking cell walls that maintain their totipotency and possess the unique ability to uptake foreign materials, including DNA, organelles, viruses, and plasmids [14]. This aspect has significantly contributed to the development of somatic hybridization and the enhancement of protoplast fusion techniques [15]. Furthermore, protoplasts provide a versatile single-cell system for in-depth studies on subcellular localization, gene function, promoter activity, protein interactions, hybridization processes, gene expression, genomics, proteomics, and metabolomics [15]. Recently, protoplast-based DNA-free genome editing systems have employed the direct delivery of ribonucleoprotein complexes, composed of Cas9 and guide RNA (gRNA), in multiple plant species [16]. For successful and efficient transformation, obtaining high-quality, concentrated protoplasts is crucial [17]. However, the effectiveness of these techniques depends on establishing a reliable protoplast separation system, which is necessary for generating an adequate number of protoplasts.

Protoplasts are isolated using either mechanical disruption or enzymatic digestion, and the process includes both isolation and purification to ensure high quality [9]. Specific enzymes are used to digest the cell wall, allowing for the isolation of protoplasts from plant materials [7]. The purification step commonly involves gradient centrifugation to isolate protoplasts from contaminants or placing the sample on ice to facilitate the natural settling of protoplasts. Variations in cell wall composition among different plant species and tissues necessitate optimizing enzymolysis conditions to their physiology to enhance protoplast yield [7]. Enzyme mixture, concentration, type of explant, digestion time, osmotic pressure, and purification method critically influence cell wall removal [18]. While efficient protoplast isolation protocols have been reported in model species like *Arabidopsis thaliana* [19] and *Nicotiana benthamiana* [20] and many other plant species, such as *Camellia oleifera* [21] and grapevine [22], *Populus simonii* × *P. nigra* [7]. However, protocols for woody plants were less established due to challenges such as thick epidermal layers, phenolic compound release, low protoplast yield, and poor viability. Adding polyvinylpyrrolidone (PVP-40) to the enzyme solution prevented phenolic oxidation, protecting cells and enhancing protoplast yield and viability [8]. In the *Vaccinium* genus, only a handful of studies have explored protoplast-based methods, mostly in *V. corymbosum* [17].

Furthermore, transformation efficiency in a PEG–mediated system is affected by factors including the concentrations of PEG and DNA, the density of protoplasts, and the length of the transformation process [21]. PEG facilitates the entry of foreign DNA or RNA into protoplasts by interacting with the cell membrane, enabling transformation in protoplasts [23]. PEG-mediated transformation has been successfully applied to establish transient transformation in a range of plants, including *Camellia oleifera* [21], *V. corymbosum* [17], *Glycine max* [24], and *Ginkgo biloba* [25].

This study reports the first robust protocol for isolating viable mesophyll protoplasts from young leaves of in vitro*-grown V. membranaceum* and achieving PEG-mediated transient gene expression using PVP-40. Although similar approaches have been reported in other *Vaccinium* species, the distinct leaf tissue composition, variation in phenolic content, and species-specific enzymatic responses in black huckleberry required customized optimization. By systematically optimizing enzyme composition, digestion time, and mannitol concentration, and including PVP-40 to limit phenolic oxidation, we achieved protoplast yield and viability higher than previously reported in blueberry and other *Vaccinium* species. GFP-based assays confirmed efficient transient expression, providing a platform for functional genomics and CRISPR/Cas9-mediated genome editing in this species and other related plant species.

## Materials and methods

### Experimental plant material and associated culture environments

**Fig. 1:**
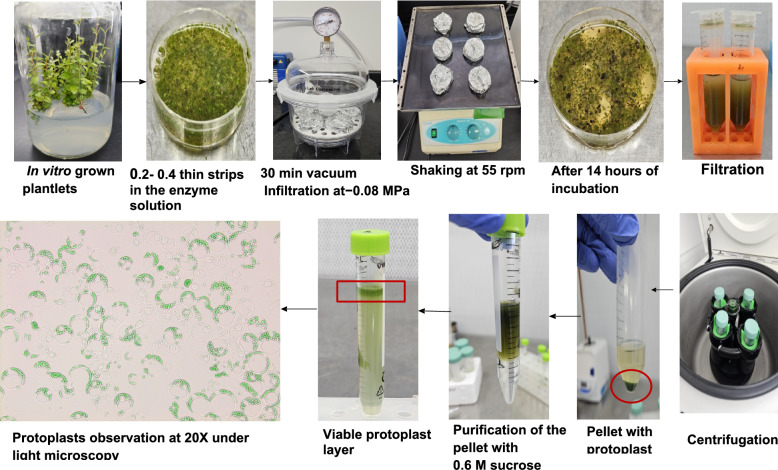
Optimized mesophyll protoplast isolation and purification procedure in *V. membranaceum*

Nodal segments from greenhouse-grown *V. membranaceum* plants of the clone ‘VM31C’ [2] were used in this study. These plants were grown and kept at the St. John’s Research and Development Centre, Agriculture and Agri-Food Canada, in St. John’s, Newfoundland and Labrador, Canada [3]. The collected explants were surface sterilized and subsequently cultured in 175-mL glass baby food jars (Sigma Chemical Co., St. Louis, MO) containing the modified Debnath and McRae’s berry basal medium (BM) (Table 1) [26], and 0, 2.3, 4.6, 9.1 μM zeatin [27]. The medium was adjusted to pH 5.0 and sterilized by autoclaving at 121 °C for 20 min. The cultures were grown in a chamber at 20 ± 2 °C with 16 h of light per day from cool white, fluorescent lamps providing 90 µmol m⁻^2^ s⁻^1^ (Conviron, Winnipeg, Manitoba, Canada) [3, 28, 29]. For each treatment, three jars were used, each with five explants. Data for direct multiple shoot bud formation was taken after 4 weeks of culture (Fig. 1). In vitro-grown plantlets were transferred to fresh medium every four weeks and kept continuously to provide leaf material for protoplast isolation (Fig. 1). Young fully expanded leaves from the shoot tips of 3- to 4-week-old in vitro-grown plantlets were used as explants for mesophyll protoplast isolation.

**Table 1:** Composition, storage conditions, and functions of media and solutions used for protoplast isolation, purification, and transformation in *V. membranaceum*

Culture media/solution name	Compositions of media/solution	Storage conditions	Function
BM	Berry basal media [26]; pH 5.0; autoclaved	Room temperature (RT)	Shoot proliferation
Enzymatic solution	Cellulase R-10 (1.0–2.5%, Plant Media, USA), hemicellulase (0.5–1.5%, Sigma-Aldrich, Canada), macerozyme R-10 (0.5–1.5%, Plant Media, USA), and pectinase (1.0–2.0%, Sigma, USA)	Freshly prepared, dark	Cell wall digestion
Modified digestion buffer	20 mmol L⁻^1^ MES–KOH (Sigma, USA), 1.0 mmol L⁻^1^ KH₂PO₄ (Sigma, USA), 1.0 mmol L⁻^1^ KNO₃ (Sigma, USA), 20 mmol L⁻^1^ KCl (Sigma, USA), 7.48 mmol L⁻^1^ CaCl₂ (Sigma, USA), 5.0 mmol L⁻^1^ sodium citrate, 0.2–0.7 mol L⁻^1^ mannitol (Sigma, USA), 0.5–2% (w/v) polyvinylpyrrolidone (PVP-40, Sigma, USA), and 0.3% (w/v) bovine serum albumin (BSA; Sigma, USA)	Freshly prepared	Cell wall digestion
W5 solution	154 mM NaCl, 125 mM CaCl2, 5 mM KCl, 2 mM MES; pH 5.6	4^0^C	Protoplast purification
Sucrose-MES solution	0.6 M sucrose and 2 mM MES·H₂O; pH 5.6	4^0^C	Protoplast purification
MMG	0.4 M Mannitol, 15 mM MgCl_2_, 4 mM MES; pH 5.6	4^0^C	Protoplast purification
LB agar media	10 g L^−1^tryptone, 5 g L^−1^yeast extract, 10 g L^−1^NaCl, 15 g L^−1^agar, supplemented with 100 mg L^−1^ kanamycin sulfate and 25 mg L^−1^ chloramphenicol; autoclaved	4^0^C	Bacteria culture
WI solution	4 mM MES, 0.4 M mannitol, 20 mM KCl, 0.22 μm filtered	4^0^C	Resuspended protoplasts after transfection
PEG–Ca^2^⁺ buffer	PEG 4000 (Sigma-Aldrich, USA), 0.2 M mannitol, and 100 mM CaCl₂·2H₂O; 0.45 µm	Freshly prepared	Plasmid transformation

### Mesophyll protoplast isolation

Fresh enzyme solutions were prepared immediately prior to mesophyll protoplast isolation to preserve enzymatic activity. To achieve optimal protoplast yield from young, fresh leaves of 3-4-week-old in vitro-grown *V. membranaceum* plantlets, the enzymatic solutions were dissolved in a modified digestion buffer (Table 1), and the pH of the enzymatic solution was optimized to 5.2, 5.4, 5.6, or 5.8. PVP-40 was added to prevent oxidation of phenolic compounds, which could compromise protoplast viability. The enzyme solution was incubated at 55 °C for 15 min to activate and fully dissolve the enzymes. It was then cooled to room temperature and sterilized by filtration through a 0.22 μm Millipore syringe filter (Millex®, Ireland) before immediate use. Freshly sliced leaf tissues were submerged in 10 mL of the enzyme solution in sterile 60 × 15 mm Petri dishes (Fisherbrand™, USA). Vacuum infiltration was performed at − 0.08 MPa for 30 min in the dark to facilitate enzyme penetration (Fig. 1). Subsequently, the tissues were incubated at 25 °C in darkness with gentle shaking at 55 rpm/min (Labnet Rocker 25, USA) for 10–18 h to complete enzymatic digestion.

### Purification of the protoplasts

For purifying the mesophyll protoplasts, the precooled W5 solution (Table 1) was added to the digestion mixture to stop enzymatic digestion, and the final volume was adjusted accordingly (Fig. 1). The mixture was filtered through sterile nylon cell strainers of various sizes (40, 70, and 100 µm) to remove undigested leaf tissue. The filtrate was collected in a 50 mL round-bottom centrifuge tube and brought up to the maximum volume. The filtrate was centrifuged at various speeds (50, 100, 150, and 200 × *g*) for 5 min at 4 °C using a benchtop centrifuge (Thermo Scientific™ Sorvall™ ST8R, Canada), followed by gentle removal of the supernatant. The protoplast pellet was gently resuspended in 10 mL of pre-chilled W5 buffer and centrifuged once more. Following removal of the supernatant, the purified protoplasts were resuspended in 2 mL of W5 buffer for subsequent applications. The resuspended protoplasts were carefully layered over 8 mL of sucrose-MES solution (Table 1) in a 15 mL centrifuge tube, then centrifuged at 1000 × *g* for 5 min at 4 °C (Fig. 1). Following centrifugation, the protoplast layer formed at the interface between the W5 and sucrose solutions was carefully collected and transferred to a new 15 mL centrifuge tube. Subsequently, 5 mL of W5 solution was added to wash the protoplasts, followed by centrifugation at 100 × *g* for 4 min at 4 °C. The supernatant was discarded, and the washing step was repeated twice. Finally, the sedimented protoplasts were resuspended in 2 mL of MMG (Table 1) solution, and the yield and viability were determined.

### Evaluation of mesophyll protoplast isolation efficiency and viability rates

Protoplast concentration and viability were determined using a double-chamber hemocytometer (XB.K.25;; QiuJing, Shanghai, China) and a fluorescence microscope (Olympus BX43, Japan). Viability was assessed by staining with 0.2% fluorescein diacetate (FDA, Sigma-Aldrich® Co). FDA (2 mg/mL in acetone) was used to stain viable protoplasts, with 10 µL of protoplasts mixed with 1 µL of 0.2% FDA solution and incubated at 25 °C in darkness for 2 min [24]. Protoplasts exhibiting green fluorescence were observed using an Olympus BX43 fluorescence microscope (Olympus BX43, Japan) at an excitation wavelength of 488 nm and an emission wavelength of 530 nm, equipped with a digital camera (Leica DFC420, Leica, Germany). Viable protoplasts exhibited green fluorescence. Counts were performed in at least three fields for each sample. Protoplast yield and viability were calculated as follows: protoplast yield (protoplasts per g of fresh weight) = protoplast number / fresh weight of leaves; protoplast viability (%) = (fluorescent protoplasts / total protoplasts) × 100%.

### Plasmid preparation

PLCS101 (Plasmid #209355, Addgene) encodes enhanced GFP, allowing efficient expression in plant protoplasts. For PEG-mediated protoplast transfection, *Escherichia coli* was cultured on LB agar media (Table 1) and incubated at 37 °C for 16 h. Single colonies were selected and transferred to liquid LB medium, followed by incubation at 37 °C with shaking at 200 rpm for 16 h. Plasmid DNA was extracted with the Qiagen Plasmid Mini Kit I (Cat. No. D6943, USA). Concentrations were quantified, and aliquots were stored at – 20 °C for later use.

### PEG-mediated protoplast transformation

**Fig. 2:**
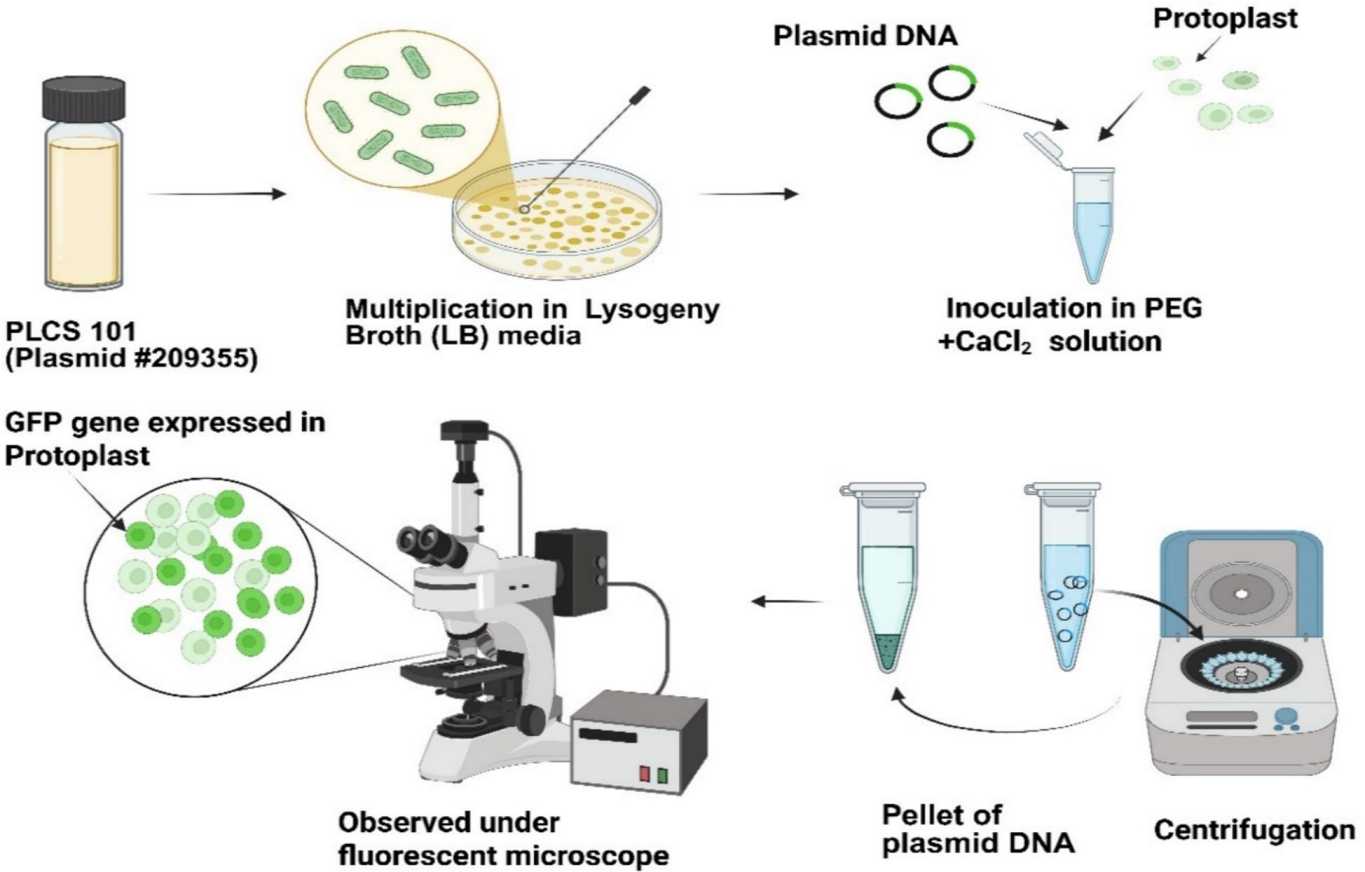
Schematic figure of an optimized PEG-mediated gene transfer protocol for improved transfection efficiency

Mesophyll Protoplasts of *V. membranaceum* were transformed using a modified PEG–Ca^2^ [30]. After quantifying yield and further purification, the protoplast suspension in W5 solution was centrifuged at 100 × *g* for 2 min at room temperature. The supernatant was gently discarded, and the pellet resuspended in 5 mL of fresh W5 solution (Fig. 2). The suspension was incubated on ice for 30 min. Afterwards, the supernatant was carefully removed, and the protoplasts were resuspended in chilled MMG solution at a final density of 2 × 10^5^ cells mL⁻^1^. A 200 µL aliquot of purified protoplasts (~ 2 × 10^4^ cells) was combined with varying amounts of PLCS101 plasmid DNA (10, 15, 20, 25, 30, 35, and 40 µg; Plasmid #209,355, Addgene) in a 1.5 mL tube, followed by an equal volume of PEG–Ca^2^⁺ transfection buffer (Table 1), sterilized by filtration through a 0.45 µm membrane. Different concentrations of PEG 4000 (0, 10, 20, 30, 40, and 50%) were tested to improve transformation efficiency. The mixture was gently inverted and incubated in the dark at room temperature for 10–35 min. Transformation was halted by adding twice the volume of W5 solution, followed by centrifugation at 100 × *g* for 2 min. The supernatant was discarded, the wash repeated once, and protoplasts resuspended in 1 mL WI solution (Table 1). They were incubated overnight (10 h) in 2% BSA-coated 6-well plates at 23 °C in the dark. GFP expression was visualized using an Olympus BX43 microscope (excitation 488 nm, emission 530 nm). Transformation efficiency was determined by calculating the percentage of green fluorescent protoplasts out of the total protoplasts observed in the field, using the formula: transformation efficiency (%) = (number of fluorescent protoplasts / total protoplasts) × 100%.

### Subcellular localization

After the transformation, protoplasts were incubated for 12–14 h in WI solution to allow for gene expression. To visualize nuclear localization, protoplasts were stained with 1 µg mL⁻^1^ 4',6-diamidino-2-phenylindole (DAPI, Sigma-Aldrich, USA) in Phosphate-Buffered Saline (PBS) for 10 min, followed by three washes with PBS [24]. GFP-tagged protein localization was observed with an Olympus BX43 fluorescence microscope, detecting GFP at 488 nm excitation and 530 nm emission, and DAPI at 358 nm excitation and 461 nm emission.

### Statistical analysis

Statistical analyses were performed using IBM SPSS Statistics for Windows, version 29.0 (SPSS Inc., Chicago, IL, USA). One-way ANOVA was conducted, followed by Duncan’s multiple range test (DMRT) for post hoc comparisons. Experiments involving variable adjustments were repeated at least three times. Data are expressed as mean ± standard error (SE) from three biological replicates. Means sharing the same letter are not significantly different. Differences were considered statistically significant at *p* < 0.05.

## Results

### Multiple shoot bud initiation and proliferation

**Fig. 3:**
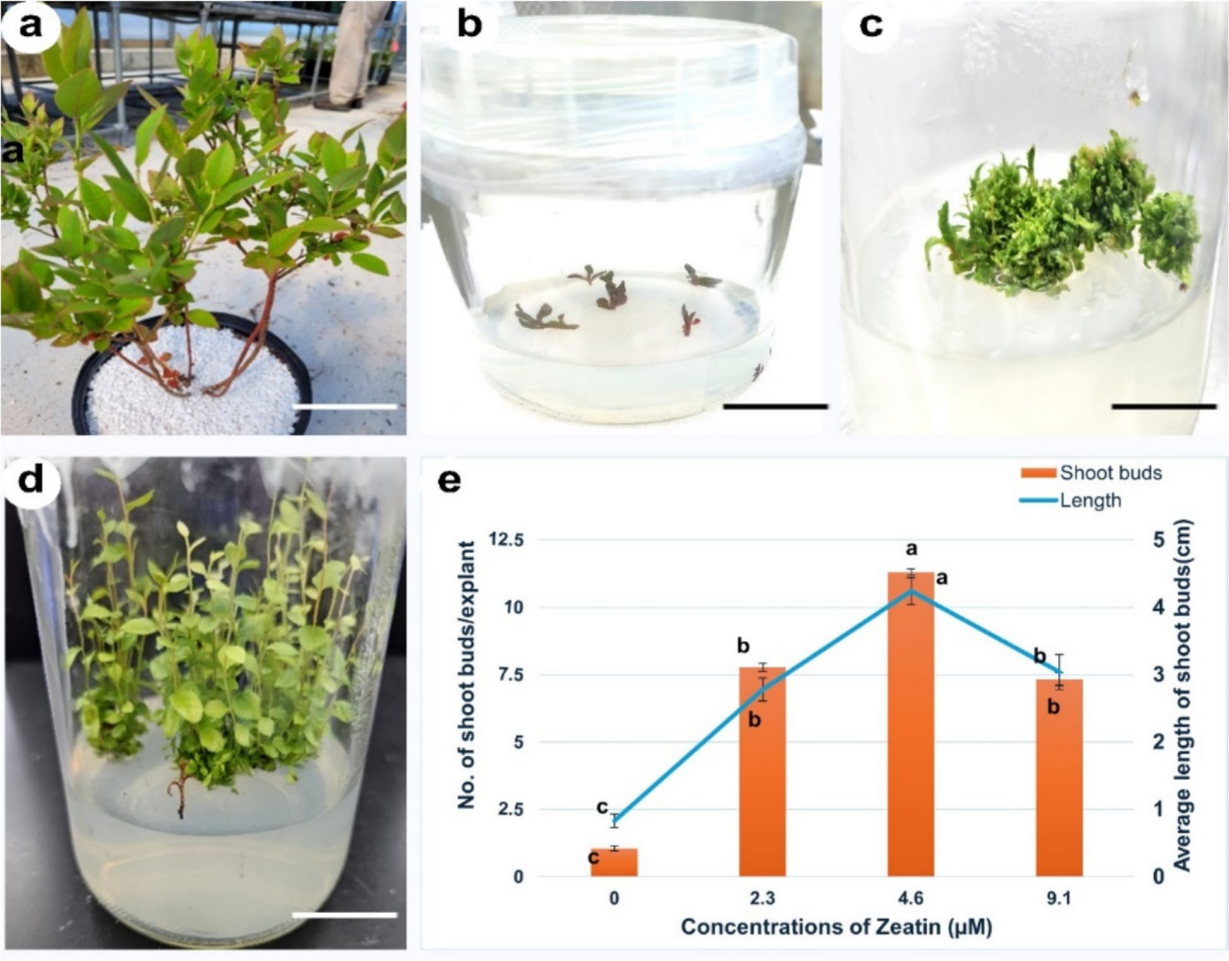
The effect of zeatin on the initiation and proliferation of shoot buds in *V. membranaceum*, **a** Donor *V. membranaceum* plants grown in a green greenhouse, **b** inoculation of nodal segments in berry basal medium (BM), **c** shoot proliferation after 2 weeks of culture, **d** shoot proliferation after 4 weeks of culture, and e) Effects of different Zeatin concentrations on shoot bud proliferation and elongation in *V. membranaceum*. Scale bar = 3 cm

For shoot multiplication, individual adventitious shoots measuring 1–1.5 cm were excised from greenhouse-grown *V. membranaceum* plants. Nodal explants were cultured on berry basal medium (BM) supplemented with four different zeatin concentrations, which significantly promoted multiple shoot formation within four weeks (Fig. 3a–d). Subsequent subculturing led to the elongation of axillary shoots, reaching lengths of 3–4 cm within four weeks. Variations in zeatin concentration (0, 2.3, 4.6, and 9.1 µM) markedly influenced both the frequency and number of shoot buds formed. The treatment containing 4.6 µM Zeatin demonstrated the most pronounced morphogenic response, yielding the maximum average number of shoots per explant (11.30 ± 0.12), the greatest average shoot length (4.24 ± 0.20 cm), after 4 weeks of culture (Fig. 3e). Conversely, explants maintained on the basal MS medium devoid of cytokinins exhibited no shoot initiation even after four weeks, thereby affirming the pivotal role of exogenous cytokinins, particularly Zeatin, in facilitating multiple shoot proliferation. This protocol provides the optimal young leaf material required for the subsequent protoplast isolation.

### Optimization of PVP-40 for mesophyll protoplast yield and viability

To develop an efficient mesophyll protoplast isolation and transient expression system in *V. membranaceum*, the selection of leaf material is essential for maximizing protoplast yield. Young leaves from 3 to 4-week-old in vitro-grown plantlets were selected for protoplast isolation due to their high cellular activity and enzymatic digestion responsiveness [14]. Mesophyll protoplasts of *V. membranaceum* were isolated using 1% cellulase, 0.5% hemicellulase, 0.5% macerozyme, and 1% pectinase with PVP-40 included at 0, 0.5, 1, and 2% (w/v) to optimize protoplast yield and viability Table 2.

**Table 2:** Effect of PVP-40 on mesophyll protoplast yield and viability in *V. membranaceum*

PVP-40 (% w/v)	Total protoplast yield (× 10⁶ g⁻^1^)	Total viable protoplast yield (× 10⁶ g⁻^1^)	Viability (%)
0 (no PVP)	0.02 ± 0.00^c^	0.00 ± 0.00^d^	0.0 ± 0.00 ^d^
0.5	0.10 ± 0.01^b^	0.02 ± 0.00^c^	15.45 ± 0.37^c^
1.0	0.13 ± 0.14^a^	0.09 ± 0.02^a^	65.99 ± 0.03^a^
2.0	0.08 ± 0.31^b^	0.01 ± 0.22^b^	7.12 ± 0.20^b^

*Data represents the mean ± standard error of three biological replicates (n = 3). Different letters within a column indicate significant differences (Duncan’s multiple range test, *p* < 0.05)

Without PVP-40, protoplast yield was minimal (0.02 ± 0.00 × 10⁶ g⁻^1^) with no viable cells (0.0 ± 0.0%). Inclusion of 0.5% PVP-40 increased yield (0.10 ± 0.01 × 10⁶ g⁻^1^) and viability (15.45 ± 0.37%), whereas 1% PVP-40 provided the highest yield and viability (0.13 ± 0.14 × 10⁶ g⁻^1^; 65.99 ± 0.03%). At 2% PVP-40, viable protoplasts decreased (0.08 ± 0.31 × 10⁶ g⁻^1^; 7.12 ± 0.20%), indicating that excessive PVP-40 negatively affects cell integrity.

These results demonstrate that 1% PVP-40 is optimal for enhancing mesophyll protoplast yield and viability, highlighting its role in minimizing phenolic-induced damage during enzymatic digestion**.**

### Optimization of enzymatic combinations for mesophyll protoplast isolation in *V. membranaceum*

**Fig. 4:**
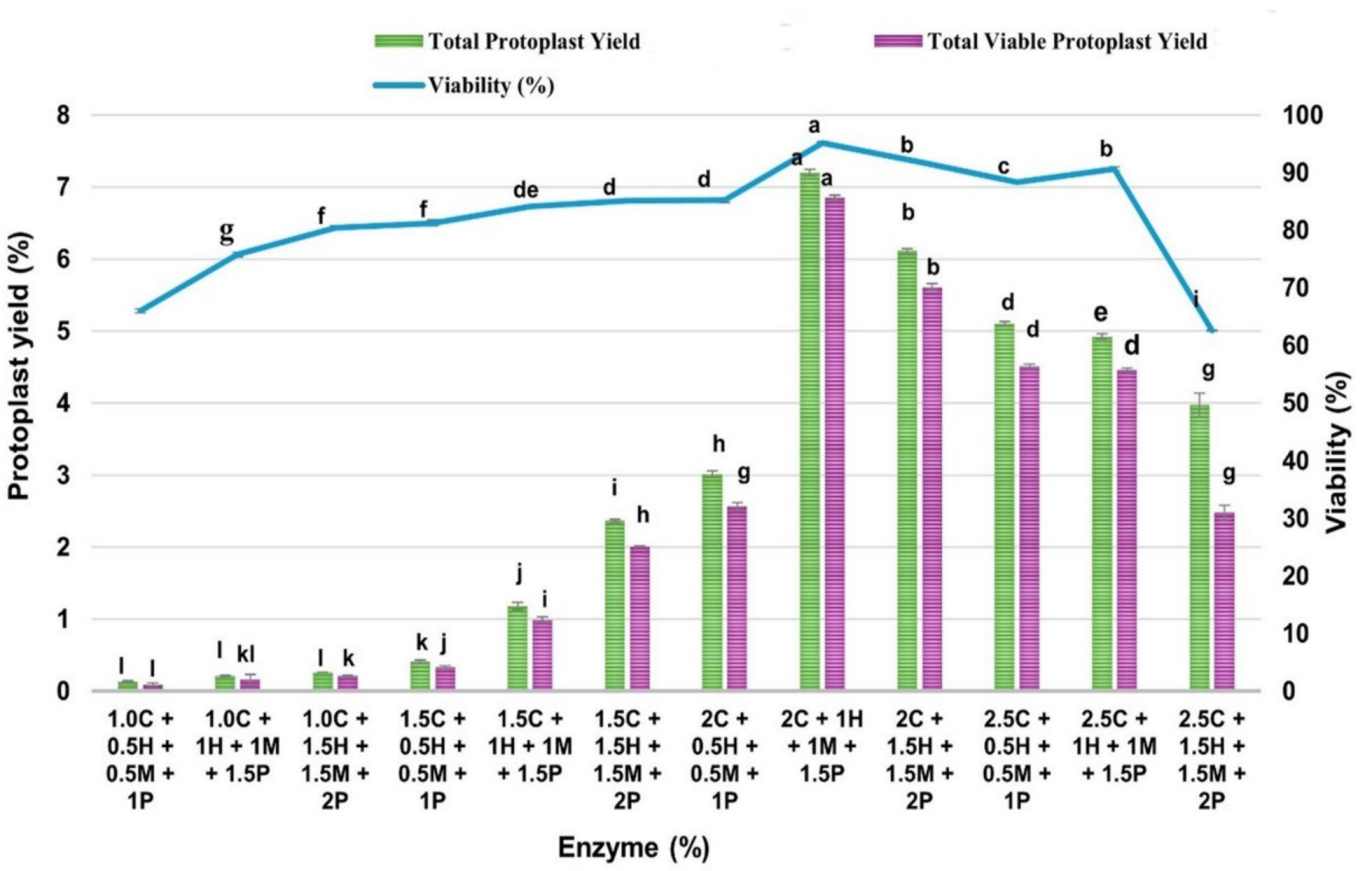
Effects of enzyme combinations with 1% PVP-40 on mesophyll protoplast yield and viability from in vitro-grown *V. membranaceum* leaves. Here, C = Cellulase, H = Hemicellulase, M = Macerozyme, P = Pectinase. Values are mean ± SE (n = 3); different letters indicate significant differences (Duncan’s multiple range test, *p* < 0.05)

**Fig. 5:**
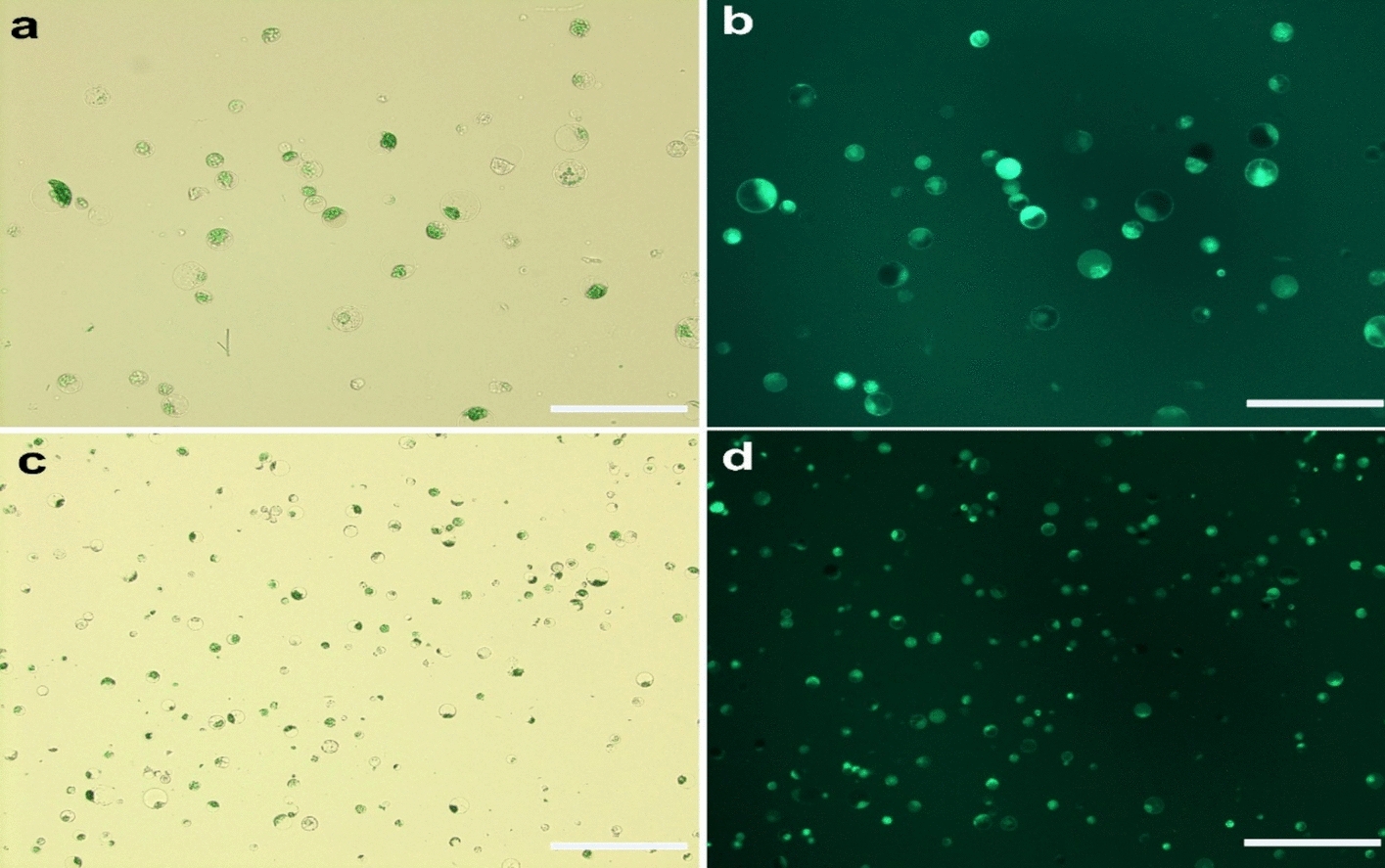
Protoplasts were isolated from leaves of in vitro-grown *V. membranaceum* plantlets. **a**, **c** Protoplast morphology was observed under light microscopy at 20× and 10× magnifications, respectively. **b**, **d** Viable protoplasts stained with fluorescein diacetate (FDA) and visualized under a fluorescence microscope (excitation 488 nm, emission 530 nm). Scale bar = 20 µm

To optimize enzymatic conditions for mesophyll protoplast isolation, twelve combinations of cellulase (1.0–2.5%), hemicellulase (0.5–1.5%), macerozyme (0.5–1.5%), and pectinase (1.0–2.0%) were tested in triplicate, each supplemented with 1% PVP-40 (Fig. 4). The lowest protoplast yield was observed in the enzyme combination comprising 1% cellulase R-10, 0.5% hemicellulase, 0.5% macerozyme, and 1% pectinase (Treatment no. 1), producing an average total yield of only 0.13 × 10^6^ protoplasts g^−1^ fresh weight (FW), with a viable yield of 0.09 × 10^6^ protoplasts g^−1^ FW and a mean viability of 65.99%. A moderate increase in the concentrations of hemicellulase (1.0%), macerozyme (1.0%), and pectinase (1.0%) in Treatment 2 resulted in a significant improvement, yielding 0.21 × 10^6^ protoplasts g^−1^ FW and enhancing viability to 75.76%. Further optimization with 1.5% cellulase, 1.0% hemicellulase, 1.0% macerozyme, and 1.5% pectinase (Treatment 4) led to a marked increase in protoplast yield (1.18 × 10^6^ protoplasts g^−1^ FW and viability (84.14%). The optimal yield and viability were achieved with Treatment 8, consisting of 2% cellulase, 1% hemicellulase, 1% macerozyme, and 1.5% pectinase. This combination yielded 7.20 × 10^6^ protoplasts g^−1^ FW, with a viable yield of 6.85 × 10^6^ protoplasts g^−1^ FW and a viability rate of 95.10% (Fig. 5a–d). However, enzyme concentrations beyond this level (Treatments 9–12) led to a decline in both yield and viability, indicating possible cytotoxic effects or excessive cell wall degradation. Overall, the combination of 2% cellulase, 1% hemicellulase, 1% macerozyme, and 1.5% pectinase yielded the highest protoplast recovery and viability, establishing it as the most effective condition for protoplast isolation in *V. membranaceum.*

### Optimization of mannitol concentration to maximize mesophyll protoplast yield and viability

**Fig. 6:**
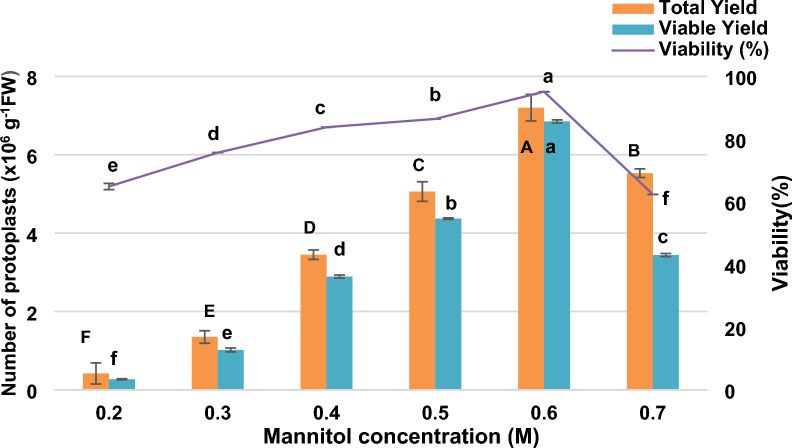
Protoplast yield and viability of *V. membranaceum* at different mannitol concentrations. The values represent the mean ± standard error of the mean (n = 3), with different letters denoting statistically significant differences at *p* < 0.05

**Fig. 7:**
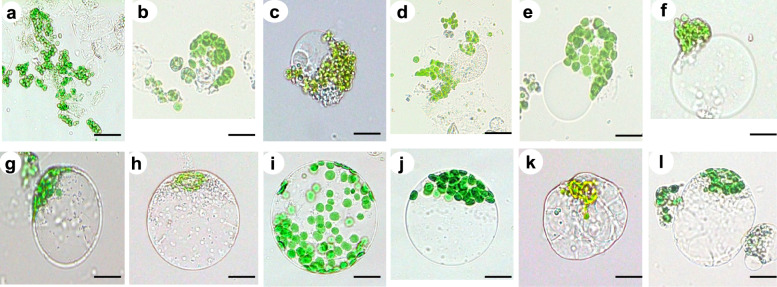
Protoplast morphology under different mannitol concentrations (0.2–0.7 M) in *V. membranaceum*. Protoplast condition at 0.2 M mannitol (**a**, **b**), 0.3 M (**c**, **d**), 0.4 M (**e**, **f**), 0.5 M (**g**, **h**), 0.6 M (**i**, **j**), and 0.7 M (**k**, **l**). Scale bar = 10 µm

Mannitol is widely used as an osmotic stabilizer in protoplast isolation buffers to maintain osmotic balance and prevent cell lysis during enzymatic digestion. To determine the optimal osmotic conditions for mesophyll protoplast isolation in *V. membranaceum*, mannitol concentrations ranging from 0.2 to 0.7 M were evaluated for their effects on yield and viability (Fig. 6). The lowest protoplast yields were recorded at 0.2 and 0.3 M, averaging 0.42 × 10⁶ and 1.35 × 10⁶ protoplasts g^−1^ FW respectively, likely due to insufficient osmotic stabilization that caused cell swelling, rupture, and accumulation of lysed protoplast debris. Yields improved markedly at 0.4 M and 0.5 M, reaching 3.45 × 10⁶ and 5.06 × 10⁶ protoplasts g^−1^ FW, respectively. While Morphological integrity was preserved only at 0.5 M (Fig. 7g, h) and 0.6 M (Fig. 7i, j) mannitol. The highest total yield (7.20 × 10⁶ protoplasts g^−1^ FW), total viable protoplast yield (6.85 × 10⁶ protoplasts g^−1^ FW), and viability (95.10%) were achieved at 0.6 M. However, 0.7 M mannitol led to a decline in both yield (5.53 × 10⁶ protoplasts g^−1^ FW) and viability (3.44 × 10⁶ protoplasts g^−1^ FW and 62.35%), likely due to osmotic stress and cellular dehydration (Fig. 7k–l). These results indicate that 0.6 M mannitol provides the most effective balance between yield and viability and is therefore optimal for protoplast isolation in *V. membranaceum.*

### Influence of enzyme solutions on mesophyll protoplast isolation efficiency in *V. membranaceum*

**Fig. 8:**
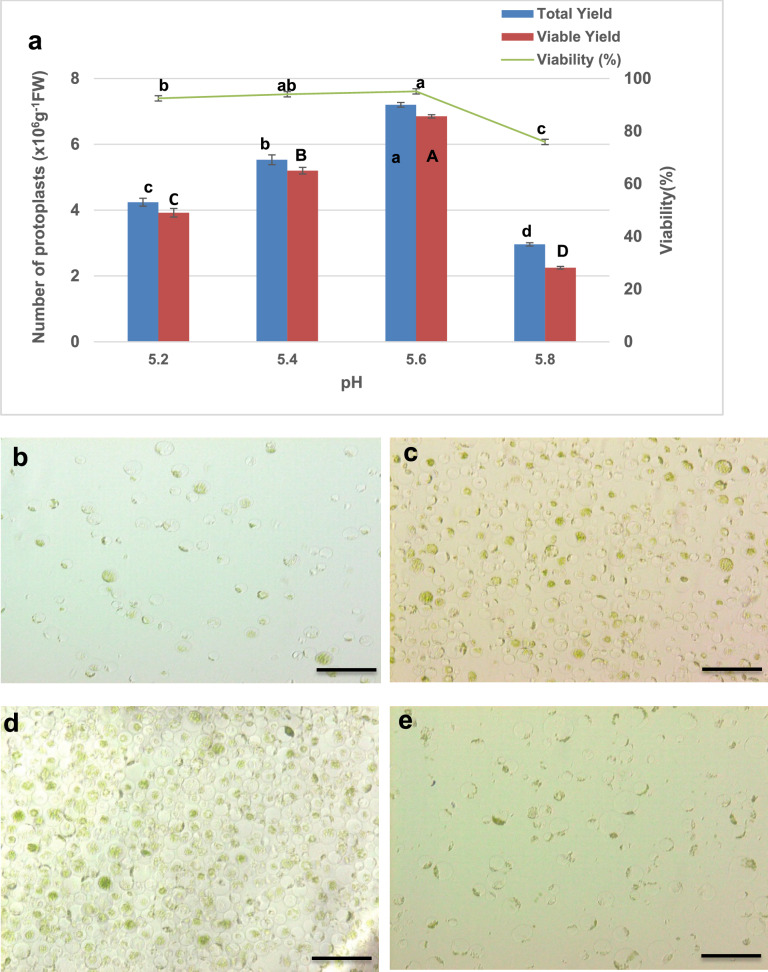
Effect of pH on mesophyll protoplast isolation from *V. membranaceum*. **a** Effect of pH on total protoplast yield, viable yield, and viability in *V. membranaceum*. Values represent the mean ± standard deviation of three biological replicates (n = 3). Statistical significance was assessed by one-way ANOVA with Duncan’s multiple range test at *p* < 0.05. Different letters denote significant differences among pH treatments. **b**–**e** Microscopic images of protoplasts isolated at pH 5.2 (**b**), 5.4 (**c**), 5.6 (**d**), and 5.8 (**e**), captured at 20× magnification. Scale bar = 50 µm

Enzyme solution pH adjustment significantly impacted mesophyll protoplast isolation efficiency from *V. membranaceum* leaves. A pH range of 5.2, 5.4, 5.6, and 5.8 was tested to determine the optimal condition (Fig. 8a). The optimal pH was determined to be 5.6, which yielded the highest total protoplast count (7.20 × 10⁶ protoplasts g^−1^ FW), viable protoplast count (6.85 × 10⁶ protoplasts g^−1^ FW), and cell viability (95.10%) (Fig. 8d). At pH 5.4, slightly lower yet comparable yields were observed (total: 5.53 × 10⁶ protoplasts g^−1^ FW; viable: 5.20 × 10⁶ protoplasts g^−1^ FW) with a viability of 94.02% (Fig. 8c). Both lower (pH 5.2) and higher (pH 5.8) pH levels significantly reduced the efficiency of protoplast isolation. Specifically, at pH 5.2, total and viable yields decreased to 4.24 × 10⁶ and 3.92 × 10⁶ protoplasts g^−1^ FW, respectively, with viability reduced to 92.46% (Fig. 8b). The most pronounced decline occurred at pH 5.8, where total and viable yields dropped sharply to 2.96 × 10⁶ and 2.25 × 10⁶ protoplasts g^−1^ FW, respectively, alongside a substantial viability reduction to 75.90% (Fig. 8e). These results indicate that deviations from the optimal pH, particularly towards alkaline conditions, adversely affect enzymatic cell wall degradation or protoplast integrity, establishing pH 5.6 as the optimal condition for efficient protoplast isolation in *V. membranaceum*.

### Impact of enzymolysis duration on mesophyll protoplast yield and viability

**Fig. 9:**
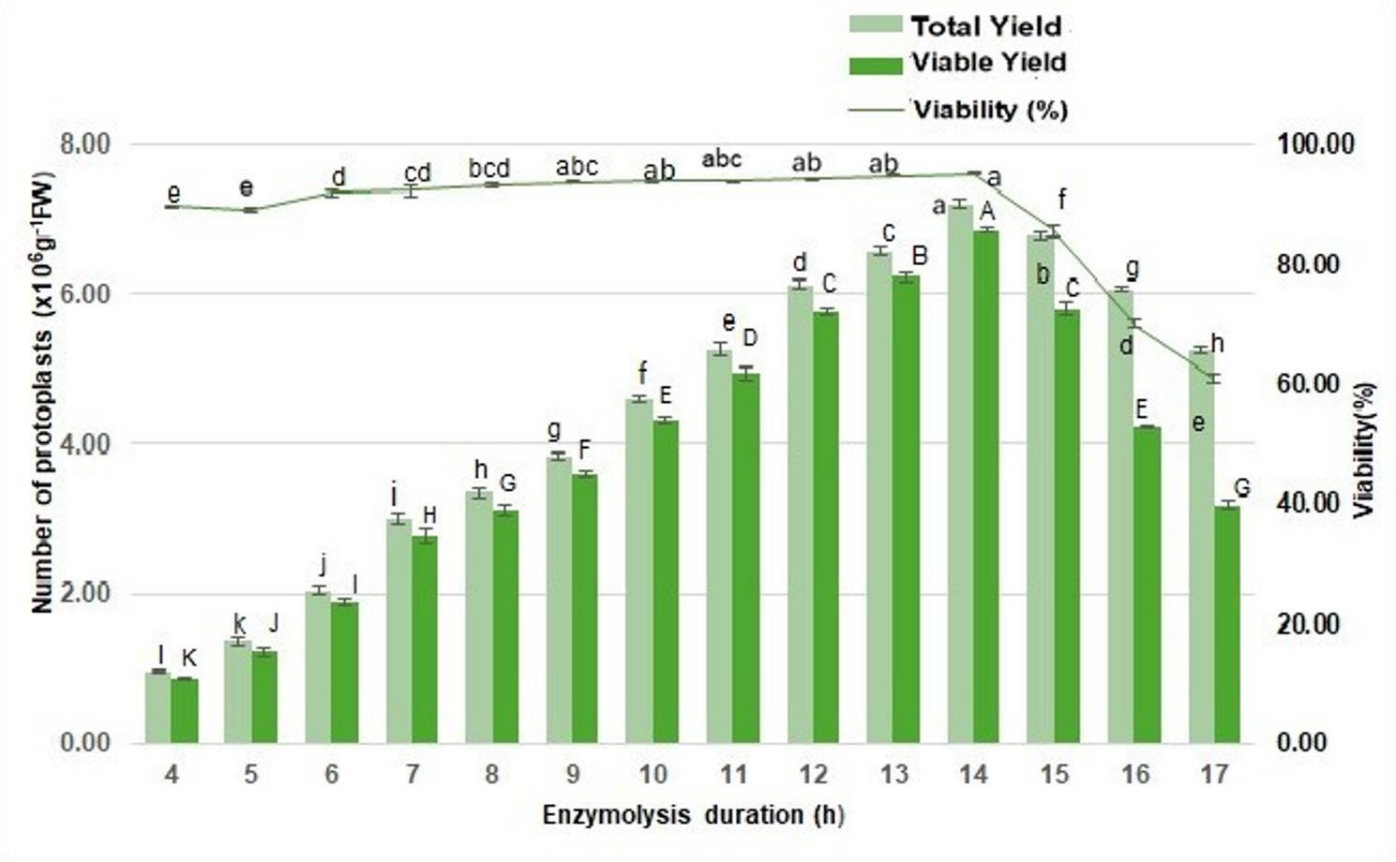
Protoplast yield and survival rate of *V. membranaceum* at various enzymatic digestion durations. Data are expressed as mean ± standard error of the mean (n = 3), with different letters signifying statistically significant differences at *p* < 0.05

The influence of enzymolysis duration on mesophyll protoplast yield and viability in *V. membranaceum* was evaluated by incubating young, fresh leaves from in vitro-grown plantlets in the selected enzyme mixture (2% cellulase, 1% hemicellulase, 1% macerozyme, and 1.5% pectinase) for periods ranging from 4 to 17 h (Fig. 9). At 4 h, protoplast recovery was minimal, with a mean yield of 0.97 × 10⁶ protoplasts g^−1^ FW and a viability of 89.72%, suggesting incomplete cell wall degradation. From 4 to 14 h, both protoplast yield and viability gradually increased, indicating improved cell wall hydrolysis. Notably, the 14-h enzymolysis period resulted in the highest protoplast yield of 7.20 × 10⁶ protoplasts g^−1^ FW, and a viable yield of 6.85 × 10⁶ protoplasts g^−1^ FW, with a viability rate of 95.10%, optimizing digestion and plasma membrane preservation (Fig. 9). In contrast, prolonging enzymolysis beyond 14 h resulted in a significant decrease in yield and viability, with 6.06 × 10⁶ and 5.25 × 10⁶ protoplasts g^−1^ FW and viabilities of 70.03% and 60.79% at 16 and 17 h, respectively. These findings suggested that 14 h is the optimal enzymatic digestion time for maximizing protoplast recovery from in vitro*-*grown *V. membranaceum* leaves.

### Impact of nylon membrane mesh size on protoplast purification

**Fig. 10:**
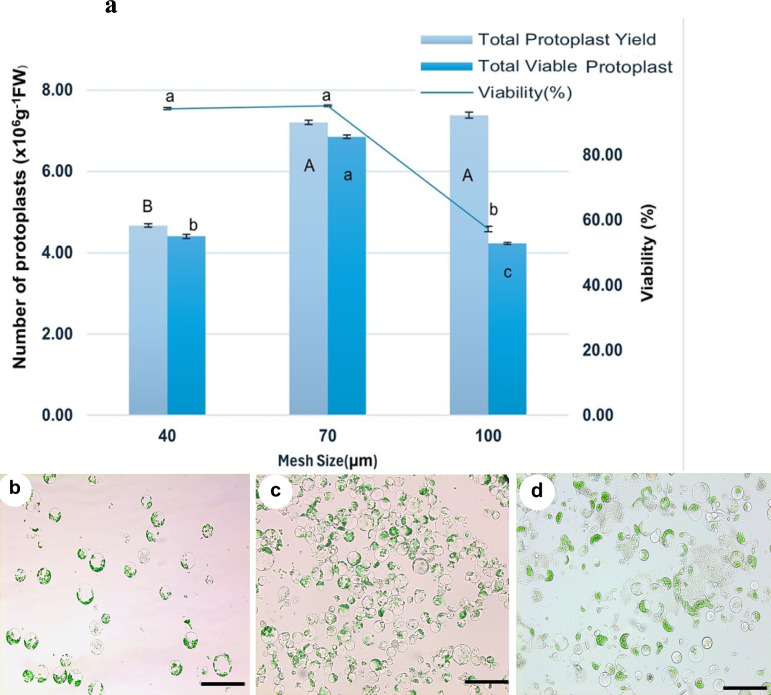
Effect of nylon mesh size on protoplast purification in *V. membranaceum*. **a** Protoplast yield and viability after filtration through 40, 70, and 100 µm nylon meshes. Values represent mean ± standard error (n = 3), with different letters indicating statistically significant differences at *p* < 0.05. **b**–**d** microscopic images of protoplasts obtained after filtration using **b** 40 µm, **c** 70 µm, and **d** 100 µm mesh. Scale bar = 50 µm

Following 14 h of digestion in the dark at room temperature with 2% cellulase, 1% hemicellulase, 1% macerozyme, and 1.5% pectinase in 0.6 M mannitol and 1%PVP-40, crude protoplasts were filtered through nylon membranes of varying mesh sizes (Fig. 10). Protoplast diameters ranged between 10 and 35 µm, with a predominant size near 20 µm, as determined by eyepiece micrometry. To evaluate the impact of nylon membrane mesh size on protoplast purification, filters with 40, 70, and 100 μm pore sizes were tested (Fig. 10a). Filtration using a 40 µm mesh significantly restricted protoplast passage, resulting in a reduced yield of 4.66 × 10⁶ protoplasts g^−1^ FW, although cell viability remained relatively high at 94.29% (Fig. 10b). Moreover, filtration through a 70 µm nylon mesh yielded the highest protoplast recovery (7.20 × 10⁶ protoplasts g^−1^ FW) and viability (95.10%), while maintaining minimal contamination with cellular debris (Fig. 10c). The 100 µm mesh facilitated greater protoplast passage (7.38 × 10⁶ protoplasts g⁻^1^ FW but was associated with increased contamination by cellular debris, leading to reduced filtrate purity and a subsequent decline in viability to 57.24% (Fig. 10d). These results indicate that the 70 µm nylon mesh strikes the optimal balance between protoplast recovery and filtrate purity, making it the most suitable pore size for effective protoplast purification in *V. membranaceum* isolation protocols.

### Effect of centrifugation speed on mesophyll protoplast yield and viability

**Fig. 11:**
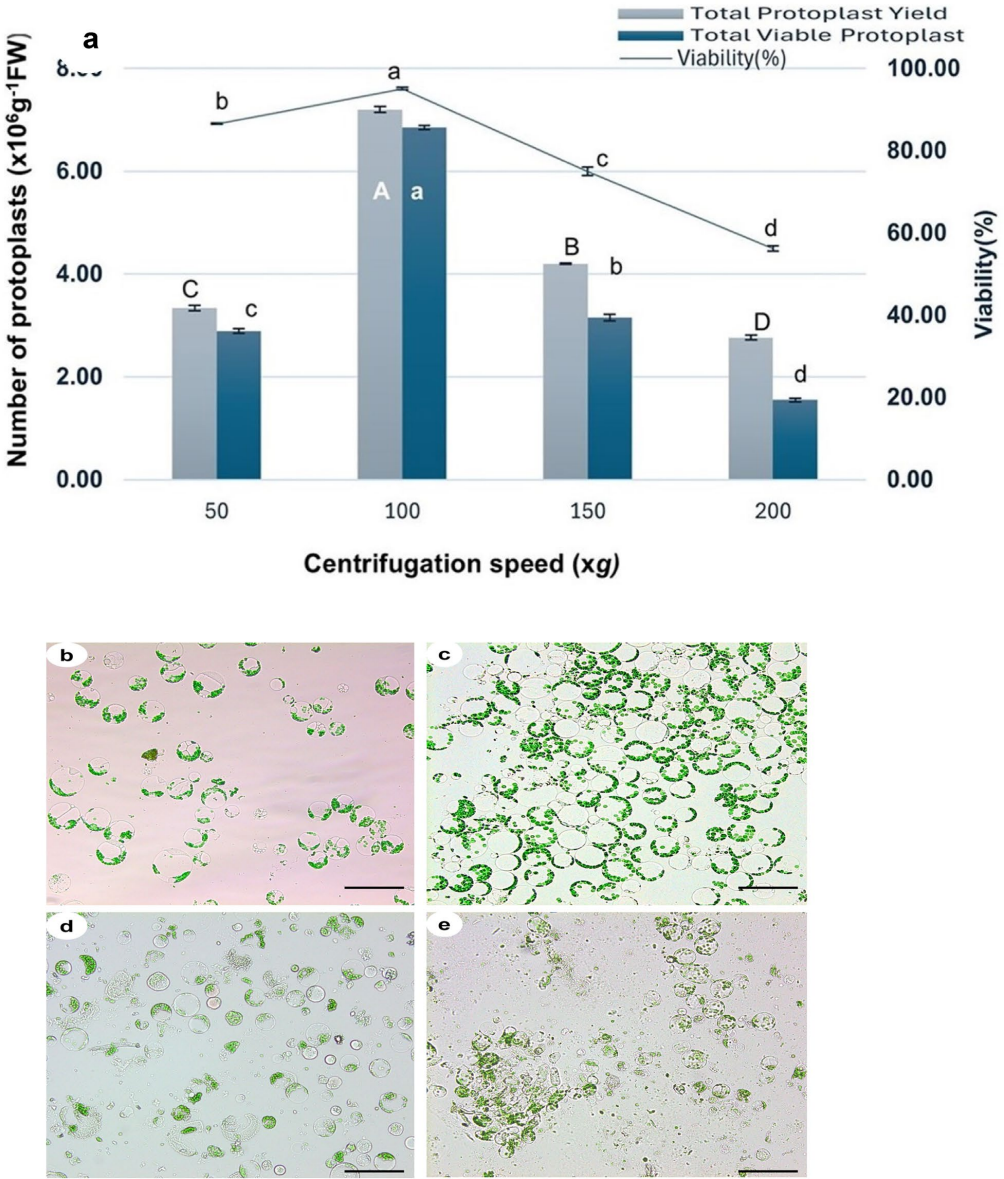
Influence of centrifugation speed on *V. membranaceum* protoplast yield and viability. **a** Total yield, viable yield, and viability (%) of protoplasts isolated at different centrifugation speeds (50–200 × *g*). Values represent the mean ± SD of three biological replicates. Letters indicate significant differences among treatments at *p* < 0.05. **b**–**e** Microscopic observation of protoplasts after centrifugation at **b** 50 × *g*, **c** 60 × *g*, **d** 80 × *g*, and **e** 100 × *g*. Scale bar = 50 μm

The digestion mixture, containing 2% cellulase R-10, 1% hemicellulase, 1% macerozyme R-10, and 1.5% pectinase in 0.6 M mannitol and 1% PVP-40, was incubated for 14 h, then filtered through a 70 µm mesh. Protoplast purification was assessed by centrifuging at 50, 100, 150, and 200 × *g* for 5 min at 4 °C. Protoplast yield and viability of the lower pellets initially rose but declined as centrifugation speed increased (Fig. 11a). At 50 × *g*, the yield was lower (3.34 × 10⁶ protoplasts g^−1^ FW) due to incomplete sedimentation, while viability remained relatively high (86.52%), indicating minimal physical stress under these conditions (Fig. 11b). The optimal centrifugal condition for protoplast recovery and cell viability was 100 × *g*, where the yield reached 7.20 × 10⁶ protoplasts g^−1^ FW, viable yield was 6.85 × 10⁶ protoplasts g^−1^ FW, and viability peaked at 95.10% (Fig. 11c). Increasing the speed to 150 × *g* caused a decline in viability (74.97%) and a reduction in yield (4.20 × 10⁶ protoplasts g^−1^ FW), indicating mechanical damage due to elevated centrifugal stress (Fig. 11d). At 200 × *g*, viability dropped further to 56.20%, and yield decreased to 2.76 × 10⁶ protoplasts g^−1^ FW, likely due to plasma membrane disruption (Fig. 11e). These findings highlight the importance of centrifugal force in protoplast isolation, with 100 × g being the optimal speed for maximizing yield and viability while minimizing damage.

In summary, mesophyll protoplasts were effectively isolated with high yield and viability after 14 h of enzymatic digestion using 2% cellulase R-10, 1% hemicellulase, 1% macerozyme R-10, and 1.5% pectinase in 0.6 M mannitol and 1% PVP-40. The 70 µm nylon mesh and centrifugation at 100 × *g* achieved the highest protoplast yield (7.20 × 10⁶ protoplasts g^−1^FW) and viability (95.10%), while effectively minimizing debris contamination. This optimized protocol offers a robust platform for obtaining viable protoplasts suitable for downstream applications such as genetic transformation and physiological assays.

### Transient gene transformation efficiency in mesophyll protoplasts of *V. membranaceum*

**Fig. 12:**
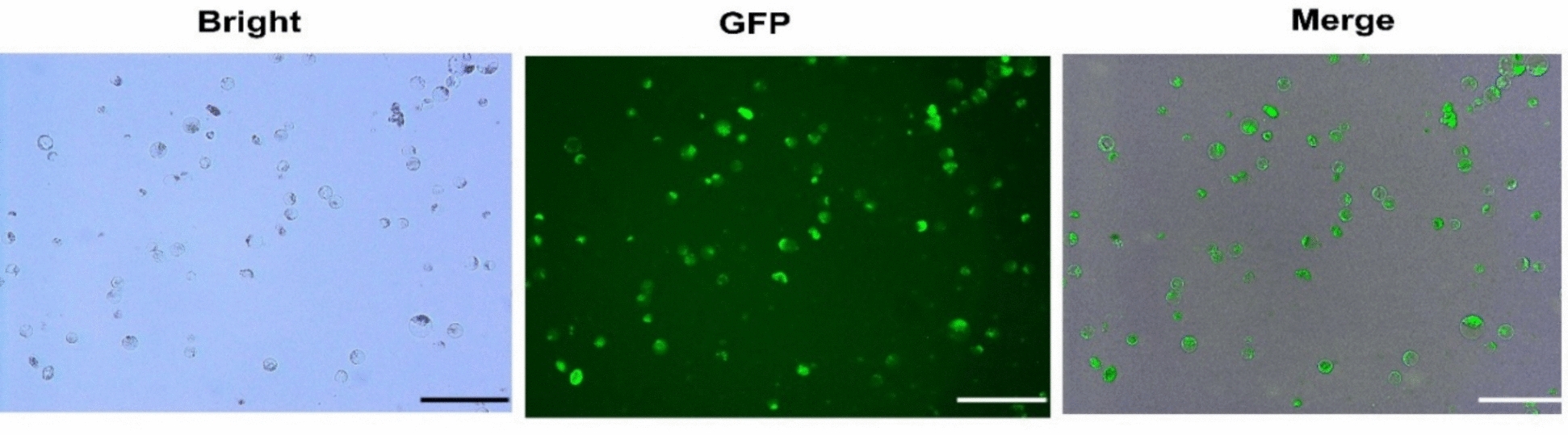
Transient expression of PLCS101 plasmid in *V. membranaceum* protoplasts. Bright-field, fluorescence, and merged images confirm successful transient expression of the GFP gene. Scale bar = 50 µm

**Fig. 13:**
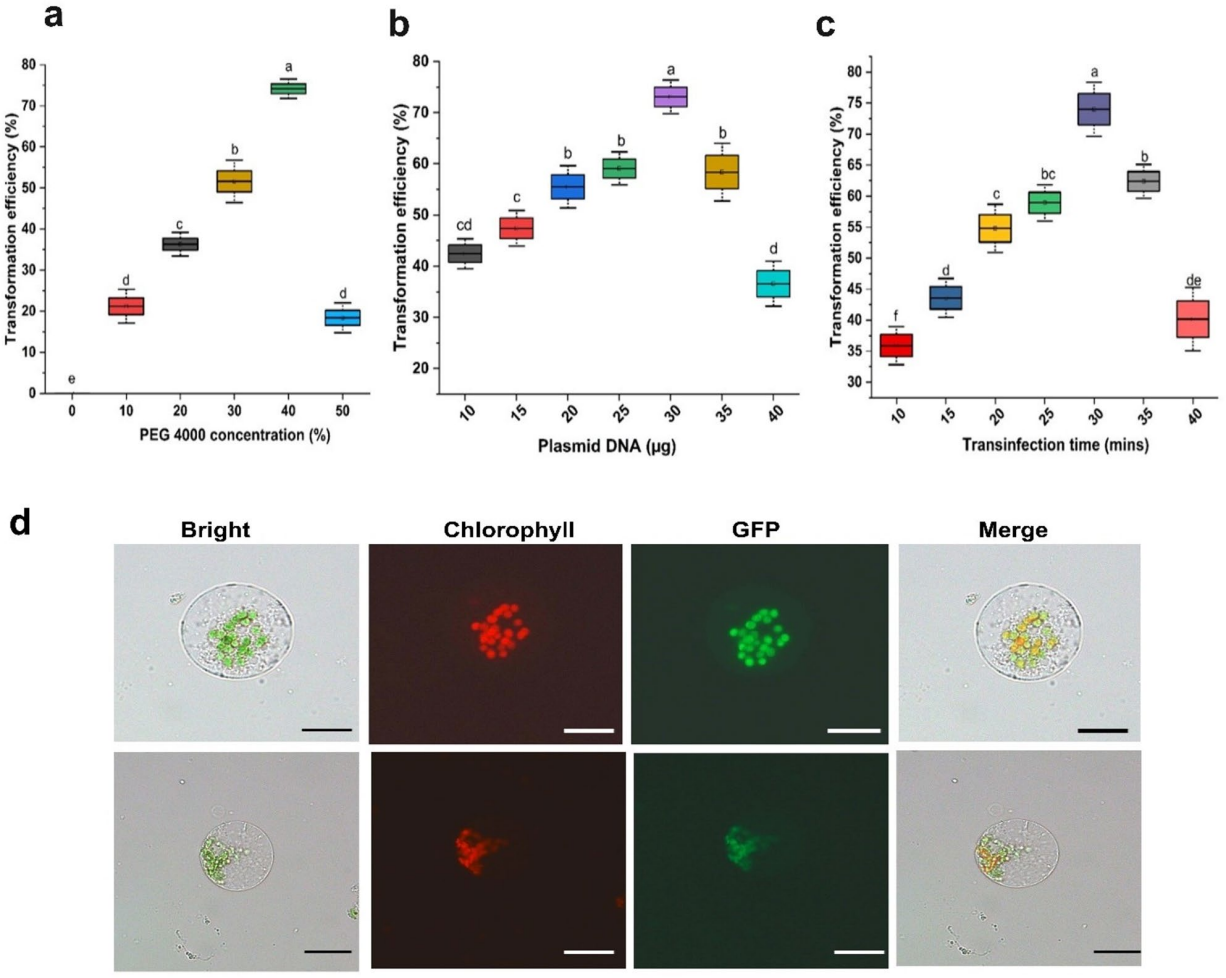
Effects of different parameters on protoplast transformation efficiency in *V. membranaceum*. **a** Effect of PEG-4000 concentration (w/v) on transformation efficiency. **b** Effects of plasmid DNA concentration (μg) on transformation efficiency (%). **c** Effects of transfection duration (min) on transformation efficiency, and **d** Bright field, chlorophyll autofluorescence, GFP fluorescence, and merged images of *V. membranaceum* protoplasts 10 h post-transfection. Merged panels display combined GFP and chlorophyll signals. Different letters indicate statistically significant differences (*p* ≤ 0.05). Scale bar = 20 µm

The effects of plasmid DNA amount, PEG 4000 concentration, and incubation time on mesophyll protoplast transformation efficiency (Fig. 12) were systematically evaluated. Varying plasmid DNA amounts from 10 to 40 µg per 100 µL protoplast suspension revealed low transformation efficiency at 10 µg (42.43%), which increased with DNA concentration and declined at higher amounts (35–40 µg) likely due to solution viscosity and osmotic stress (Fig. 13b).

Under the optimized plasmid DNA condition (30 µg), different PEG 4000 concentrations (0, 10, 20, 30, 40, and 50% w/v) were tested in 100 mM CaCl₂ and 0.2 M D-mannitol. No transformation was observed in the absence of PEG, and low efficiency (22.85%) was observed at 10% PEG. Transformation efficiency increased with PEG concentration, peaking at 40% before declining at 50% due to protoplast debris and morphological abnormalities (Fig. 13a).

Using the optimized plasmid DNA (30 µg) and PEG 4000 (40%) conditions, incubation times from 10 to 40 min were evaluated. Efficiency was lowest at 10 min (35.90%), increased with longer incubation, and declined after 30 min, likely due to PEG-induced cytotoxicity (Fig. 13c).

Finally, by combining the optimal conditions of 30 µg plasmid DNA, 40% PEG 4000, and 30-min incubation time, the highest transient transformation efficiency of 74.16% was achieved in *V. membranaceum* mesophyll protoplasts, as confirmed by strong GFP fluorescence (Fig. 13d).

### Subcellular localization of GFP-tagged protein in *V. membranaceum* protoplasts

**Fig. 14:**
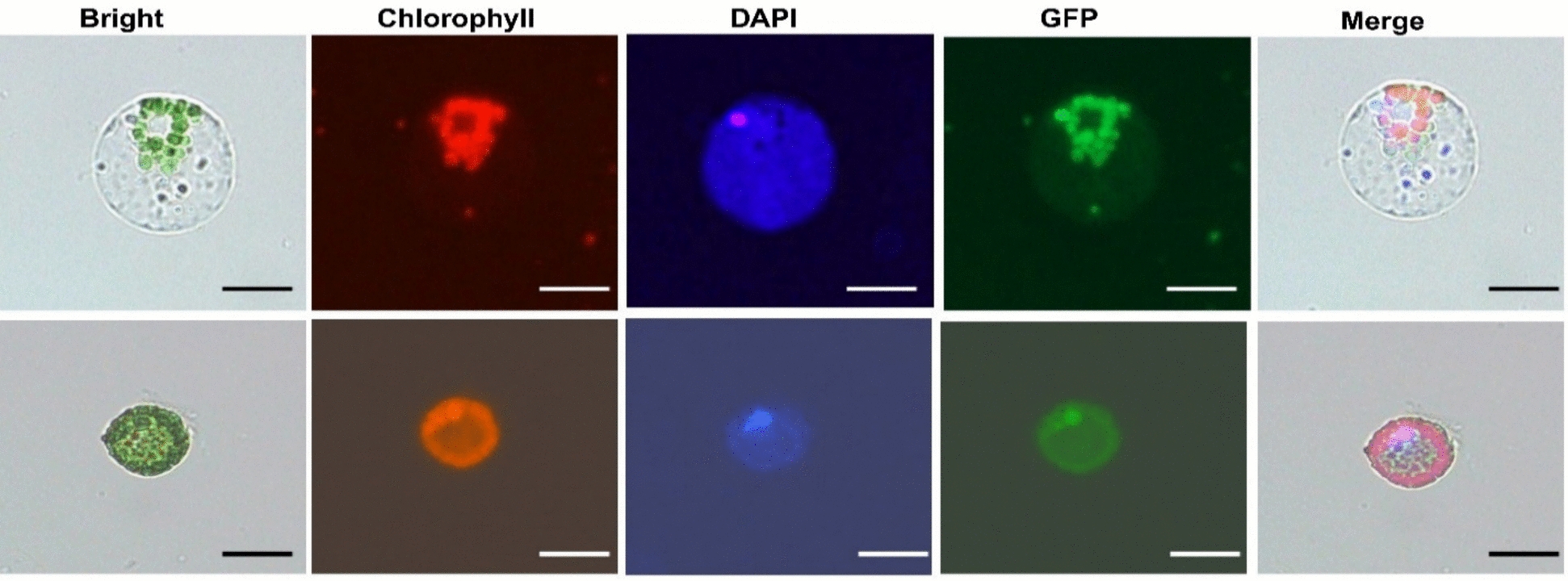
Fluorescence microscopy images illustrated the subcellular distribution of PLSC101 GFP in *V. membranaceum.* **a** The nucleus is identified by DAPI staining, and chloroplasts are indicated by chlorophyll autofluorescence. Signals from DAPI, GFP, and chlorophyll were observed simultaneously in individual protoplasts. Scale bar = 20 µm

To validate the transient gene expression system, we examined whether the introduced GFP gene was localized correctly within protoplasts derived from leaves of in vitro-grown *V. membranaceum* (Fig. 14). Protoplasts were transfected with the PLCS101 plasmid containing GFP under the UBQ10 promoter using PEG–Ca^2^⁺ at 40% PEG, 30 μg DNA, and 30 min incubation. The UBQ10 promoter caused GFP to be expressed throughout the protoplasts, visible after 12 h using a fluorescence microscope. GFP fluorescence in transfected protoplasts was detected in both the cytoplasm and nucleus, confirmed by co-localization with the DAPI nuclear stain (Fig. 14). These results demonstrate the effectiveness of the transient gene expression system for subcellular localization studies in *V. membranaceum* leaf-derived protoplasts.

## Discussion

Viable protoplasts are essential for advancing plant physiology and genetic enhancement [9]. Although protoplast isolation and transient transformation have been achieved in banana [31], *Populus* [7], and *Ginkgo biloba* [25], these processes remain challenging in woody horticultural species due to thick epidermal layers, dense cuticles, and tissue recalcitrance [10]. In *V. membranaceum*, incorporation of 1% PVP-40 into the enzyme solution markedly reduced phenolic oxidation, resulting in a substantial increase in mesophyll protoplast yield and viability. The highly viable, actively dividing protoplasts obtained are appropriate for efficient PEG-mediated transient gene expression. Notably, the mesophyll protoplast yield obtained here exceeded that previously reported *V. corymbosum* (5.95 × 10^4^ protoplasts g⁻^1^ FW), demonstrating the effectiveness of PVP-40 in enhancing recovery. This protocol provides a reliable platform for the isolation of uniform, aseptic protoplasts and enables functional genomics and gene function studies in *V. membranaceum*.

Free protoplasts, lacking a cell wall, are prone to osmotic imbalance, leading to swelling, bursting, or shrinkage; thus, appropriate osmotic stabilizers are essential to maintain pressure equilibrium during isolation [20]. Mannitol functions as an effective osmotic by maintaining pressure equilibrium and preventing cell rupture during enzymatic digestion [32]. Reported optimal concentrations for maintaining protoplast integrity vary among species, typically ranging from 0.2 to 0.7 M (Fig. 6). In this study, both hypoosmotic and hyperosmotic conditions negatively affected mesophyll protoplast isolation from leaves of in vitro-grown *V. membranaceum*. Protoplasts showed swelling and lysis at 0.2–0.4 M mannitol, while concentrations above 0.7 M caused plasmolysis and reduced transient transformation efficiency. An optimal concentration of 0.6 M mannitol yielded the highest stability and viability (Fig. 7a–l), maintaining osmotic balance, preserving morphology, and supporting efficient PEG-mediated transformation. This result is consistent with findings for sugarcane [11], whereas optimal concentrations of 0.8 M, 0.5 M, and 0.4 M have been reported for *V. corymbosum* [17], *Solanum lycopersicum* [33], and *Glycine max* [24], respectively. These comparisons highlight the species-specific nature of osmotic regulation during protoplast isolation and the need for empirical optimization across plant systems.

Protoplast isolation relies on enzymatic degradation of the cell wall, whose cellulose, hemicellulose, and pectin composition varies among species, requiring a species-specific enzyme combination for efficient release [11, 24]. Both enzyme type and concentration must be optimized, as they directly influence yield and viability—excessive levels may increase yield but compromise membrane integrity, reducing viability [22]. Balancing enzyme concentration with digestion time is therefore critical for optimal protoplast isolation [21]. Phenolic oxidation, a major limitation in woody plants, can cause browning, enzyme inhibition, and cell death. In this study, 1% PVP-40 markedly improved mesophyll protoplast yield and viability in *V. membranaceum* (0.13 × 10⁶ g^−1^ FW, 65.99%; Table 2), whereas lower (0.5%) or higher (2%) concentrations reduced viability. Similar improvements have been reported in holm oak [8]. By comparison, DTT inhibited polyphenol oxidation in *Quercus acutissima* leaves but failed to produce adequate protoplast numbers or viability, limiting its suitability for subsequent culture [34]. Using an optimized enzyme mixture of 2% Cellulase R-10, 1% hemicellulase, 1% Macerozyme R-10, and 1.5% pectinase, *V. membranaceum* produced the highest total protoplast yield (7.6 × 10⁶ protoplasts g^−1^ FW) with 95.02% viability. Similarly, in *Eucommia ulmoides*, the combination of 2.5% Cellulase R-10, 2.5% pectinase, 0.5% hemicellulase, and 0.6% Macerozyme R-10 yielded 12.00 × 10^5^ total and 11.33 × 10^5^ live protoplasts g^−1^ FW [35]. In *V. corymbosum*, an enzyme solution containing 1% cellulase, 1.5% macerozyme, and 0.3% pectinase after 28 h incubation produced 5.95 × 10^4^ protoplasts g^−1^ FW from in vitro leaves [17], whereas in Mortiño, a 5 h digestion with 2.5% cellulase, 3% macerozyme, and 0.3% pectinase yielded 1.05 × 10^5^ protoplasts g^−1^ FW from callus [36]. For *Ginkgo biloba*, treatment with 3% Cellulase R-10 and 0.25% Pectolase Y-23 for 3 h yielded 2.18 × 10⁷ protoplasts g^−1^ FW with 95.37% viability [25]. Collectively, these results highlight the crucial role of optimizing enzyme composition and concentration in conjunction with 1% PVP-40 to maximize mesophyll protoplast yield and viability in *V. membranaceum*.

The pH of the enzymatic solution is a critical factor in mesophyll protoplast isolation, affecting both enzyme activity and cell viability. In this study, pH 5.6 was optimal for *V. membranaceum*, consistent with reports on *Capsicum annuum* [37] and *Ricinus communis* [38], and slightly lower than the optimal pH of 5.7 reported for *V. corymbosum* [7]. At pH 5.8, protoplast yield and viability declined, despite the absence of visible cell damage, suggesting reduced efficiency due to suboptimal enzymatic activity or pH-induced physiological stress affecting membrane integrity [39]. Similar declines at pH 5.8 have been observed in sweet cherry [39]. Conversely, pH 5.8 has been optimal for species such as *Dianthus caryophyllus* [40], *Glycine max* [24], and kiwifruit [13], further emphasizing the species-specific nature of enzymatic pH requirements.

Enzymolysis time is a critical factor in protoplast isolation, as it directly influences both yield and viability [13]. Optimal digestion promotes efficient protoplast release, whereas prolonged enzymolysis can cause cell damage, leading to protoplast rupture and loss of metabolic activity [22]. In this study, protoplast yield increased progressively from 6 to 12 h, reaching a maximum at 14 h, where both yield and viability were highest. Beyond 14 h, particularly at 16 h, a significant decline in protoplast quality was observed, consistent with findings in *Eucommia ulmoides* [35]. Optimal enzymolysis durations differ among species: 12 h for *Camellia sinensis* [41], 16 h for embryogenic callus of *Vitis* cultivars [42] and *Arabidopsis thaliana* [19], and 8 h for *Dianthus caryophyllus* [40]. These results confirm that 14 h represents the optimal digestion time for *V. membranaceum*, with yield and viability declining significantly beyond this period.

Enzymatic digestion of leaf tissue produces a suspension containing intact protoplasts, undigested material, lysed cells, and debris, all of which can compromise the viability and purity of the final preparation [40]. Filtration is commonly employed to remove large contaminants, with mesh pore size being a key factor in balancing debris removal and protoplast retention. This study evaluated the effect of filter pore size on protoplast yield and integrity (Fig. 10a–d). A 40 µm mesh effectively removed debris but reduced yield by excluding larger protoplasts, whereas a 70 µm filter preserved these protoplasts while eliminating debris, resulting in improved yield and viability. These findings are consistent with previous reports in *Vitis vinifera* [42] and *Capsicum annuum* [37], where 70 µm meshes facilitated efficient isolation, and similar mesh sizes have been used in *Fagopyrum tataricum* [43] and *Argemone mexicana* [44]. In contrast, other species, including *Lactuca sativa* [45] and *Morus* spp. [46], required finer filters (30–50 µm), while *Glycine max* was successfully processed using a 100 µm mesh [24]. Collectively, these results highlight the importance of tailoring filtration parameters to species-specific morphological characteristics. For *V. membranaceum*, a 70 µm pore size provided the optimal balance between protoplast yield and integrity.

Following filtration, residual cell debris was further removed through stepwise centrifugation, enhancing protoplast purity (Fig. 11). In this study, centrifugation at 100 × *g* for 5 min at 4 °C effectively isolated viable *V. membranaceum* protoplasts, maintaining over 95% viability while minimizing mechanical damage (Fig. 11c). These conditions are consistent with protocols for *Ginkgo biloba* [25] and *Morus* spp. [46], which also employed 100 × *g* for 5 min. Comparable forces were applied for 10 min in *Glycine max* [24], whereas a gentler speed of 60 × *g* was used for *V. corymbosum* [17], and a higher speed of 200 × *g* for 4 min was required in *Actinidia deliciosa* [13]. These observations underscore the necessity of optimizing centrifugation parameters for each species to maximize protoplast yield and viability.

Removal of the cell wall renders protoplasts competent for exogenous DNA uptake, enabling efficient gene transfer. Polyethylene glycol (PEG)-mediated transformation is preferred for its simplicity and effectiveness, but its success depends on PEG concentration, plasmid DNA amount, and incubation time, all of which require species-specific optimization (Fig. 12a–c).

In this study, a 40% (w/v) PEG solution combined with 30 µg of plasmid DNA yielded the highest transformation efficiency in *V. membranaceum* mesophyll protoplasts, consistent with observations in sugarcane [11]. Transformation efficiency increased with plasmid DNA up to 30 µg, beyond which it plateaued, similar to the saturation reported in oil palm at 50 µg [47]. Higher PEG concentrations (50%) reduced protoplast viability and induced cell clumping, interfering with the detection of GFP-expressing cells, as also reported in *Camellia oleifera* [21].

Incubation time was a critical factor for transformation. In *V. membranaceum*, a 30-min incubation yielded the highest transformation efficiency, whereas longer durations (> 35 min) reduced efficiency. Similar results have been reported in *V. corymbosum*, where optimal transformation was achieved with 35–40 µg plasmid DNA and 45% PEG for 35 min [17]. Optimal incubation times vary among species, including 15 min for *Brassica oleracea* [48], 20 min for *Camellia oleifera* [49], and 30 min for banana [31]. These findings highlight the necessity of optimizing PEG concentration, plasmid DNA amount, and incubation time to maximize transformation efficiency across different plant species.

GFP subcellular localization was assessed via co-localization with DAPI, a nuclear DNA–binding dye (Fig. 14). Overlapping GFP and DAPI signals confirmed GFP presence in both the cytoplasm and nucleus of *V. membranaceum* mesophyll protoplasts, demonstrating that the PLCS101 plasmid enables effective gene expression and accurate subcellular targeting. DAPI-based nuclear validation is widely used in plants, as shown in *Glycine max* [24] and *Ginkgo biloba* [12]. These results confirm the reliability of this method for evaluating protein distribution in plant protoplasts.

Optimization of osmotic balance, enzyme mix, pH, and digestion time with 1% PVP-40 significantly enhanced protoplast yield, viability, and transformation efficiency. The developed *V. membranaceum* protocol offers a reliable platform for functional genomics and enables efficient CRISPR/Cas9 RNP delivery to investigate genes such as VmANS (*Anthocyanidin synthase*) involved in anthocyanin biosynthesis.

## Conclusion

In conclusion, we report the first optimized protocol for mesophyll protoplast isolation in *V. membranaceum*, achieving high yield and viability using 1% PVP-40. Using an enzymatic mix of 2% cellulase R-10, 1% hemicellulase, 1% Macerozyme R-10, 1.5% pectinase in 0.6 M mannitol with 1% PVP-40 and a 14-h incubation, the highest yield reached 7.20 × 10⁶ protoplasts g^−1^ FW with 95.1% viability. PVP-40 was crucial in minimizing phenolic oxidation, enhancing both yield and viability. PEG-Ca^2^⁺ mediated transformation efficiency peaked at 75.1%, and nuclear localization of GFP-tagged proteins was confirmed via DAPI staining. This robust system provides a valuable platform for functional genomics, gene editing, and molecular breeding in *V. membranaceum* and other plant species.

## Data Availability

No datasets were generated or analysed during the current study.

## Abbreviations

PEG: Polyethylene Glycol
DAPI: 4′,6-Diamidino-2-phenylindole
GFP: Green fluorescent protein
BM: Berry basal medium
PVP: Polyvinylpyrrolidone
BSA: Bovine serum albumin
FDA: Fluorescein diacetate
FW: Fresh weight
