# Correction: PVP-40 mediated enhancement of mesophyll protoplast yield and viability for transient gene expression in black huckleberry

**DOI:** 10.1186/s13007-025-01488-0

**Published:** 2026-01-05

**Authors:** Sweety Majumder, Abir U. Igamberdiev, Samir C. Debnath

**Affiliations:** 1 St. John’s Research and Development Centre, Agriculture and Agri-Food Canada, St. John’s, NL Canada; 2 Department of Biology, Memorial University of Newfoundland, St. John’s, NL Canada

**Correction: Plant Methods (2025) 21:150** 10.1186/s13007-025-01471-9

The copyright holder for this article was incorrectly given as 'The Author(s) 2025'but should have been 'His Majesty the King in Right of Canada as represented bythe Minister of Agriculture and Agri-Food Canada and Sweety Majumder, Abir U.Igamberdiev 2025'.

The original [1] has been corrected.
